# The complete mitochondrial genome of Mekong tiger fish *Datnioides undecimradiatus* (Roberts & Kottelat, 1994)

**DOI:** 10.1080/23802359.2016.1168720

**Published:** 2016-07-05

**Authors:** Lei Wang, Zaizhong Chen, Jianzhong Gao, Yuming Zhao, Peiying Sun, Kai Lu

**Affiliations:** a Key Laboratory of Freshwater Fishery Germplasm Resources, Ministry of Agriculture, Shanghai, PR China;; b Shanghai Collaborative Innovation Center for Aquatic Animal Genetics and Breeding (ZF1206), Shanghai Ocean University, Shanghai, PR China

**Keywords:** Mekong tiger fish, mitogenome, next-generation sequencing

## Abstract

In this study, the complete mitogenome sequence of the Mekong tiger fish *Datnioides undecimradiatus* (Perciformes: Datnioididae) has been sequenced by next-generation sequencing method. The assembled mitogenome consisting of 16,534 bp, includes 13 protein-coding genes, 22 transfer RNAs and 2 ribosomal RNAs genes. The overall base composition of the Mekong tiger fish is 27.9% for A, 30.69% for C, 16.51% for G, 24.91% for T and show 77% identities to *Anoplocapros lenticularis*(GenBank accession no. NC011319.1). The complete mitogenome of the Mekong tiger fish provides essential and important DNA molecular data for further phylogeography and evolutionary analysis for Datnioididae family.

The Mekong tiger fish *Datnioides undecimradiatus* (Roberts & Kottelat 1994) occurs in middle and lower Mekong basin. Usually found in main streams and large tributaries, feeding on fishes and shrimps (Vidthayanon 2005). Its maximum length reached 40 cm in total length (Baird et al. 1999).

Samples of the Mekong tiger fish were imported from Vietnam, and the muscle was preserved in pure alcohol. The specimens were stored in Fish Specimens Museum in Shanghai Ocean University, the accession number is SHOU20150084001. Then, their genomic DNA was extracted from muscle by using Genomic DNA Purification Kit (GeneMark, Taichung, Taiwan). The methods for genomic DNA extraction, library construction and next-generation sequencing were followed by previous publication (Shen et al. 2014). The raw next-generation sequencing reads were de novo assembled by commercial software (Geneious V8, Auckland, New Zealand) to produce a single, circular form of complete mitogenome with about an average 338.4× coverage (13,496 out of 9,637,558, 0.0014%). The complete mitochondrial genome of the Mekong tiger fish was 16,534 bp in size (GenBank accession no. KU870663), includes 13 protein-coding genes, 22 transfer RNAs and two ribosomal RNAs genes. The overall base composition of the Mekong tiger fish is 27.9% for A, 30.69% for C, 16.51% for G, 24.91% for T and show 77% identities to *Anoplocapros lenticularis* (GenBank accession no. NC011319.1) (Figure 1).

**Figure 1.**
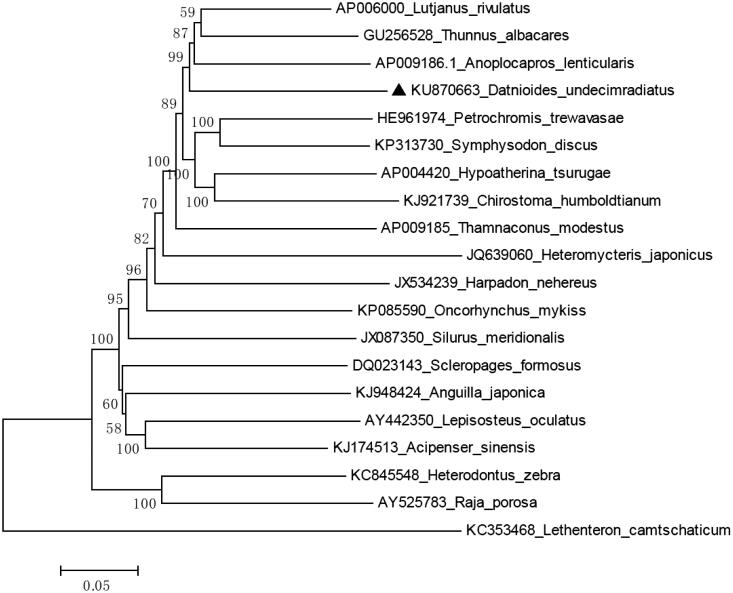
Neighbour-joining (NJ) tree of 20 species complete mitochondrial genome sequence. The phylogenetic relationships of Mekong tiger fish show 77% identities to *Anoplocapros lenticularis*.

The protein-coding and tRNA genes of the Mekong tiger fish mitogenome were predicted by using DOGMA (Wyman et al. 2004), ARWEN (Laslett & Canback 2008) and MitoAnnotator (Iwasaki et al. 2013) tools. Some ambiguous annotation sites are manual inspected. All protein-coding genes were encoded on H-strand with exception of protein-coding genes of *ND6*. All tRNA genes were encoded on H-strand with exception of *tRNA-Glu*, *tRNA-Pro*, *tRNA-Gln*, *tRNA-Ala*, *tRNA-Asn*, *tRNA-Cys*, *tRNA-Tyr* and *tRNA-Ser* (UGA). All the 13 mitochondrial protein-coding genes share the start codon ATG, except for COX1 (GTG start codon) and ATP6 (TTG start codon). The stop codon, TAA, is present in *ND1*, *COI*, *ATP8*, *ND4L*, *ND5* and *ND6*; an in complete stop codon ‘‘TA–’’ is found *inND2*, COIII and *ATP6*, and ‘‘T––’’ is found in COII,ND3, ND4 and Cytb.

The longest one is *ND5* gene (1839 bp) in all protein coding genes, whereas the shortest is *ATP8* gene (168 bp). The two ribosomal RNA genes, 12S rRNA gene (968 bp) and 16S rRNA gene (1709 bp), are located between *tRNA-Phe* and *tRNA-Leu* (UAA) and separated by *tRNA-Val*. We expect that the present result would elucidate the further phylogenetic approach among different species of Datnioididae.

To validate the phylogenetic position of *D. undecimradiatus*, MEGA6 software (Tamura et al. 2013) were used to construct a neighbour-joining (NJ) (with 1000 bootstrap replicates) containing complete mitogenomes of 20 species (Figure 1). In conclusion, the complete mitogenome of *D. undecimradiatus* provides essential and important DNA molecular data for phylogenetic and evolutionary analysis for Datnioididae.
